# Enabling and Enhancing Massive Multiple Input–Multiple Output Systems with Two-Dimensional Orthogonal Pattern Division Multiple Access

**DOI:** 10.3390/s26113491

**Published:** 2026-06-01

**Authors:** Ruimai Wang, Jianguo Yao, Yanling Shi, Ziwei Liu, Xiaodong Bai

**Affiliations:** 1 School of Communication and Information Engineering, Nanjing University of Posts and Telecommunications, Nanjing 210003, China; 1223013835@njupt.edu.cn (R.W.); ylshi@njupt.edu.cn (Y.S.); lzw@njupt.edu.cn (Z.L.); 2 School of Computer Science and Technology, Hainan University, Haikou 570228, China; xiaodongbai@hainanu.edu.cn

**Keywords:** orthogonal pattern division multiple access, massive MIMO, optimal frequency hopping patterns, multipath interference, Doppler effect

## Abstract

This paper proposes a two-dimensional orthogonal pattern division multiple access (OPDMA) technique to address key challenges in massive MIMO systems, including complex channel estimation, multipath interference, Doppler effects, and inter-antenna interference. Byleveraging optimal frequency hopping patterns with ideal autocorrelation and cross-correlation properties, constructed using a two-dimensional cyclic shift method, OPDMA eliminates the need for equalizers and channel estimation, thereby simplifying receiver design and mitigating pilot contamination. A method for constructing these patterns is introduced, based on an algebraic Costas array with a two-dimensional cyclic shift approach. The simulation results show that OPDMA significantly reduces the bit error rate (BER), simplifies system architecture, and enhances communication quality. These findings highlight OPDMA’s potential to improve performance and streamline the design of massive MIMO systems compared to traditional methods, which implies that OPDMA can be a promising low-complexity interference-suppression strategy when the optimal frequency hopping patterns design parameters match the expected Doppler shift and multipath delay.

## 1. Introduction

Massive multiple input–multiple output (MIMO) is a cornerstone technology for 5G networks. Unlike traditional MIMO, which uses a limited number of antennas, massive MIMO employs a significantly larger antenna array at the base station. Despite its potential, wireless communication channels in massive MIMO systems are susceptible to various forms of interference, such as multipath propagation and Doppler effects. Furthermore, pilot contamination can degrade channel estimation accuracy, which negatively affects system performance [1]. These challenges reduce signal reliability, increase receiver complexity, and ultimately raise implementation costs.

To address these challenges, various multiple access schemes and interference mitigation techniques have been extensively investigated in recent years. Orthogonal frequency division multiple access (OFDMA), non-orthogonal multiple access (NOMA), and sparse code multiple access (SCMA) have attracted considerable attention for improving spectral efficiency and supporting massive connectivity in wireless communication systems [2,3]. In addition, frequency hopping techniques have been widely studied because of their favorable anti-interference capability and robustness against multipath fading and Doppler effects. Nevertheless, many existing approaches still require complicated channel estimation, equalization, and interference cancellation processes, which increase implementation complexity in massive MIMO systems [4].

In this context, orthogonal pattern division multiple access (OPDMA) based on optimal frequency hopping patterns (OFHPs), which exhibits ideal auto-correlation and favorable cross-correlation properties, provides a promising alternative framework for interference suppression and reliable transmission. OFHPs possess desirable autocorrelation and cross-correlation characteristics, which can effectively reduce interference caused by multipath propagation, Doppler shifts, and multi-user access [5,6]. This paper proposes applying orthogonal pattern division multiple access in massive MIMO systems to mitigate interference caused by multipath propagation, Doppler effects, and inter-user interference. Previous works [7] established the theoretical foundation for optimal frequency hopping patterns, and [8] introduced the OPDMA concept. Building upon these foundations, this work extends OPDMA to massive MIMO architectures, effectively tackling the aforementioned challenges. By designing and allocating OFHPs, OPDMA enhances interference suppression [8]. Additionally, by eliminating the need for equalization and reducing channel estimation requirements, this approach minimizes pilot contamination, helping to maximize the potential of massive MIMO systems. Moreover, it simplifies receiver design, thereby improving overall communication quality.

This paper investigates the integration of frequency hopping communication in massive MIMO systems, focusing on the application of OPDMA using OFHPs. This work builds on the concept of Costas arrays to design OFHPs for multi-antenna massive MIMO architectures. The proposed approach is validated through practical examples and simulations.

The key contributions of this paper are as follows: (i) Introducing OPDMA, which uses OFHPs with ideal autocorrelation and cross-correlation properties to effectively mitigate interference from multipath propagation, Doppler effects, and inter-user interactions. (ii) Demonstrating how OPDMA eliminates the need for equalizers and channel estimation, thereby simplifying receiver design and reducing pilot contamination. (iii) Proposing a method to construct OFHPs using a two-dimensional cyclic shift approach based on algebraic Costas arrays. (iv) Designing OFHPs for cellular massive MIMO systems and performing simulations to evaluate their effectiveness in reducing bit error rate (BER), simplifying system architecture, and improving communication quality.

The remainder of this paper is organized as follows: Section 2 introduces the massive MIMO system model and discusses the interference issues and key challenges faced by massive MIMO systems. Section 3 provides an overview of frequency hopping technology and establishes the foundation for studying OPDMA and OFHPs. It also presents the Lempel–Greenberger (L-G) model of frequency hopping patterns based on m-sequences, which serves as a benchmark for comparison. Section 4 delves into the construction of Costas arrays and the development of OFHPs, focusing on their application in massive MIMO systems. Section 5 applies OFHP arrays to a massive MIMO cellular system, presenting simulation results for performance evaluation and comparison. Finally, Section 6 summarizes the key findings and innovations of this work.

## 2. Introduction to Massive MIMO Technology

Consider a MIMO system with *M* transmit antennas and *N* receive antennas. The transmitted signal vector is sequence $x^{T}=\left[x_{1},x_{2},\ldots ,x_{M}\right]$, and the received signal vector is sequence $y^{T}=\left[y_{1},y_{2},\ldots ,y_{N}\right]$. The received signal can be represented as follows [9]:

(1) $$y=\sqrt{p}\;Hx+n,$$

where *p* is the transmit power, H is the N×M channel matrix modeling the wireless channel effects, and n represents the noise vector.

In practical wireless environments, the channel matrix H is time-varying and affected by various propagation phenomena, which makes the signal transmission more complex.

MIMO is a core technology in 5G communication, enabling multiple input–multiple output operation through large-scale antenna arrays at both the transmitter and receiver. Multipath propagation and Doppler shifts cause signal fading and distortion, degrading signal quality and increasing bit error rates. To ensure communication quality, receivers typically use equalization techniques to mitigate multipath interference and optimize signal transmission [10].

To fully utilize massive MIMO, the receiver relies on channel estimation, often using pilot arrays. However, limited coherence intervals cause pilot reuse, leading to pilot contamination, which degrades estimation accuracy. Traditional methods handle multipath interference but struggle with Doppler shifts [11]. High receiver complexity further challenges performance. Effective mitigation of interference, pilot contamination, and complexity is key to optimizing massive MIMO.

To establish a unified modeling framework, the general MIMO matrix formulation is connected with the OPDMA waveform-based representation. The transmitted symbols in each antenna are expressed using the proposed frequency-hopping BPSK-modulated waveform. Substituting the waveform structure into the MIMO signal model yields an equivalent discrete-time representation, where the channel response is projected onto the frequency-hopping patterns space.

## 3. Overview of Frequency Hopping Communication

Modern communication systems demand enhanced resistance to interference and improved security. Frequency hopping, known for its robust anti-interference capabilities, security, speed, and reliability, is widely employed in radar and other applications [12]. The key innovation of this work lies in applying frequency hopping to massive MIMO systems, where we design two-dimensional orthogonal OFHPs tailored to the system architecture (see Section 4). This section explores the underlying principles and key technologies, providing a foundation for the OPDMA technique.

**Figure 1:**
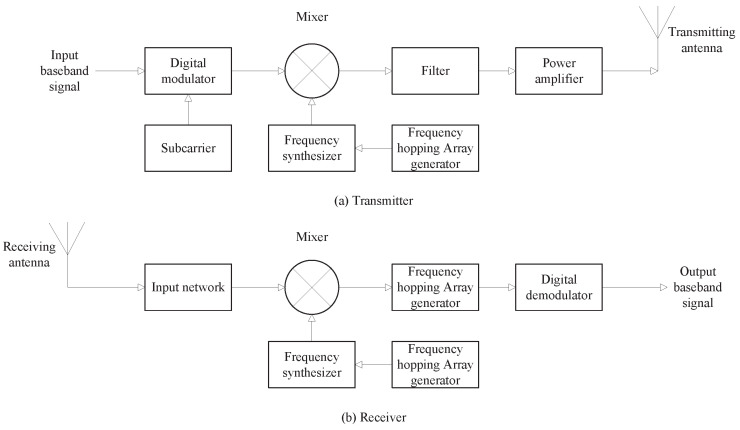
Overall transceiver structure of a frequency-hopping communication system.

Figure 1 illustrates the overall transceiver architecture of a frequency-hopping communication system, including the transmitter (a) and the receiver (b).

At the transmitter side, the input baseband signal first enters a digital modulator for modulation, where it is mapped onto a subcarrier to form a modulated signal. Meanwhile, a frequency-hopping array is generated by the frequency-hopping array generator and fed into the frequency synthesizer to produce a time-varying hopping carrier. This carrier is then mixed with the modulated signal in a mixer, enabling dynamic frequency hopping. Subsequently, the signal passes through a filter and a power amplifier before being radiated by the transmitting antenna.

At the receiver side, the received signal is captured by the receiving antenna and first processed by an input network for front-end conditioning, then sent to the mixer. Under the control of a locally frequency-hopping array, the frequency synthesizer generates a hopping carrier that matches the transmitter, enabling dehopping of the received signal [13]. After that, the signal is processed by a hopping array control and a digital demodulator, ultimately recovering the output baseband signal.

The core of this system lies in the correlation-based alignment of the frequency-hopping arrays between the transmitter and the receiver, which enables correct dehopping, demodulation, and signal recovery.

The L-G model frequency hopping patterns are known for their strong one-dimensional autocorrelation properties, and the m-sequence is one of the common solutions. These patterns are generated using *z* registers in an m-sequence generator as taps to implement frequency hopping. An *n*-stage m-sequence generator, controlled by a clock signal, produces an m-sequence of length $2^{n}-1$. As the *z*-register states continuously change, they are combined modulo 2 with a binary *z*-tuple used for user differentiation to form the frequency-hopping array. The L-G model includes $2^{z}$ frequency-hopping arrays, each with a period of $2^{n}-1$ and $2^{z}$ frequency points [14].

In addition to its structural simplicity, the L-G model exhibits favorable autocorrelation properties, which make it suitable as a baseline scheme for frequency hopping system design. However, its cross-correlation performance is relatively weak, and its frequency distribution is not perfectly uniform, which leads to increased multi-user interference and degrades system performance in terms of bit error rate (BER). Moreover, it does not strictly satisfy orthogonality requirements, which limits its effectiveness in high-density multi-user massive MIMO and high-resolution radar systems.

## 4. Construction of the Optimal Frequency Hopping Patterns

In the 1960s, John P. Costas introduced frequency-hopping signals to enhance sonar performance, leading to the Costas array, known for its ideal two-dimensional autocorrelation. Due to technological limitations, high-order Costas arrays were difficult to obtain, with a maximum order of 12 [15]. Later, Solomon W. Golomb applied finite field theory to develop the Welch and Golomb construction methods [16,17], which became widely used [7,18]. These methods are regarded as foundational constructions in array design theory. Their extensions have been further investigated in later works for application in modern wireless communication systems, including massive MIMO and multi-user multiple access scenarios. The authors of [6] introduced OFHPs, using two-dimensional cyclic shifts on Costas arrays to establish a mathematical model and analyze their algebraic structures, autocorrelation, and cross-correlation properties.

Unlike Costas arrays, which lack ideal cross-correlation properties [5,19,20], OFHPs have both ideal autocorrelation and cross-correlation. The next section explains how to design OFHPs using Golomb Costas arrays.

### 4.1. Constructing Optimal Frequency Hopping Patterns Based on Costas Arrays

Let GF(q) be a finite field, where $q=p^{m}$, with *m* as a positive integer and *p* as a prime number. Define α and β as two primitive elements in GF(q), and let C be a permutation matrix of order q−2. For C to be a Golomb Costas array, its placement function must satisfy the necessary and sufficient condition:

(2) $$y\left(k\right)=\log_{\beta }\left(1+\alpha^{k}\right),1\leq k\leq q-2.$$

When m>1, the Golomb Costas array is constructed in an extension field, where a cell at (i,j) is marked black if it satisfies $\alpha^{i}+\beta^{j}=1$. This equation represents congruence modulo f(x), where f(x) is any monic irreducible polynomial of degree *m* over GF(p).

To illustrate the construction of Golomb Costas arrays in an extension field, we use $GF(3^{2})$ as an example, where q=9, p=3, and m=2. The process begins by identifying primitive elements in $GF(3^{2})$, where multiplication follows modulo f(x). Over GF(3), there are three irreducible polynomials of degree 2: $x^{2}+1$, $x^{2}+x+2$, and $x^{2}+2x+2$. For simplicity, we choose $f\left(x\right)=x^{2}+2x+2$. Using the Euler function, ϕ(q−1)=ϕ(8)=4, we find that $GF(3^{2})$ has four primitive elements. Testing all nonzero elements helps determine the complete set of primitive elements and their powers, as shown in Table 1.

Using Table 1, the Golomb Costas array is formed with α=x and β=x+2. Based upon Equation (2), and noting that the characteristic of the finite field $GF(3^{2})$ is 3, we obtain

(3) $$\beta^{y(k)}=1+2\cdot \alpha^{k},1\leq k\leq q-2.$$

Based on Equation (4) and Table 1, the Golomb Costas array is determined to be the sequence {5,3,7,4,6,1,2}.

The Golomb Costas array has an ideal autocorrelation property, with a maximum sidelobe value of 1. Let C be a Golomb Costas array of order q−2. Define $w,s\in Z^{+}$, where $Z^{+}$ is the set of positive integers, satisfying the following equation:

(4) w·s≤q−1,

where *w* is the minimum Doppler distance between frequency hopping pattern families, *s* is the number of families, and q−1 is the maximum patterns per family.

To form a new array $C_{1}$, add an empty column to the right of C and an empty row on top. Then, shift $C_{1}$ upward by *w*, 2w, …, and (s−1)w rows to generate $C_{1}$, $C_{2}$, $C_{3}$, …, and $C_{s}$. These arrays serve as the initial frequency hopping patterns for each family.

For 1≤k≤q−1, the initial pattern shifts left by $w_{1}$, $2w_{1}$, …, and $\left(s_{1}-1\right)w_{1}$ columns, generating $s_{1}-1$ optimal patterns, each with one gap column and one gap row. The parameters $w_{1}$ and $s_{1}$ must meet the following conditions:

(5) $$w_{1}\cdot s_{1}\leq q-1,$$

where $w_{1}$ represents the minimum time-delay distance between frequency hopping patterns within the family, while $s_{1}$ indicates the number of OFHPs in the family.

Consequently, the placement function of the $l_{i}$-th optimal frequency hopping pattern in the *i*-th family is formulated as

(6) $$y_{i,l_{i}}\left(k\right)=\left(i-1\right)w+\log_{\beta }\left(1-\alpha^{\left(l_{i}-1\right)w_{1}}\alpha^{k}\right),$$

where $i=1,2,\ldots ,s;l_{i}=1,2,\ldots ,s_{1}$.

The Doppler shift is normalized by the hopping interval Δf. If it remains below the Doppler distance between two OFHPs, ideal cross-correlation holds, regardless of multipath delay. Similarly, the multipath delay is normalized by $T_{b}=1/\Delta f$. If it stays within the time-delay distance, the patterns retain ideal cross-correlation, unaffected by Doppler shift.

### 4.2. Designing Optimal Frequency Hopping Patterns for Massive MIMO Systems

First, the order of the array is set. The minimum Doppler distance w between families of OFHPs is derived from the maximum Doppler shift $d_{\max}$ (a non-negative integer), as defined in Equation (7):

(7) $$d_{\max}\leq w-1.$$

Next, the minimum time-delay distance $w_{1}$ within a family is determined by the maximum multipath delay $\tau_{\max}$ in the cell, where is a non-negative integer, as defined in Equation (8):

(8) $$\tau_{\max}\leq w_{1}-1.$$

Suppose C is a Golomb Costas array of order q−2. Using the uplink as an example, users transmit data and control signaling to the base station over wireless channels. In a massive MIMO system, let $N_{1}=4$ be the number of cells per cluster, $N_{2}=1$ represent the receiving antennas per base station, $N_{3}=1$ denote the optimal frequency hopping pattern families per antenna, $M_{1}=4$ be the users per cell, and $M_{2}=2$ signify the transmitting antennas per user. The number of optimal frequency hopping pattern families required for a cluster is determined by $N_{1}$, $N_{2}$, and $N_{3}$, as defined in Equation (9):

(9) $$s\geq N_{1}\cdot N_{2}\cdot N_{3}.$$

The number of frequency hopping patterns $s_{1}$ in each family is determined by $M_{1}$, $M_{2}$, $N_{2}$, and $N_{3}$, as defined in Equation (10):

(10) $$s_{1}\geq \frac{M_{1}\cdot M_{2}}{N_{2}\cdot N_{3}}.$$

Assume the maximum Doppler shift is $d_{\max}=1$ and the maximum delay within a cell is $\tau_{\max}=1$. The minimum Doppler distance between families is w=2, so the initial pattern $C_{1}$ shifts upward by 2 rows. Within the same family, the minimum time-delay distance is $w_{1}=2$, causing the initial pattern to shift left by 2 columns to generate the OFHPs. Thus, q−1≥max(4·2,4·2), leading to q≥8.

**Figure 2:**
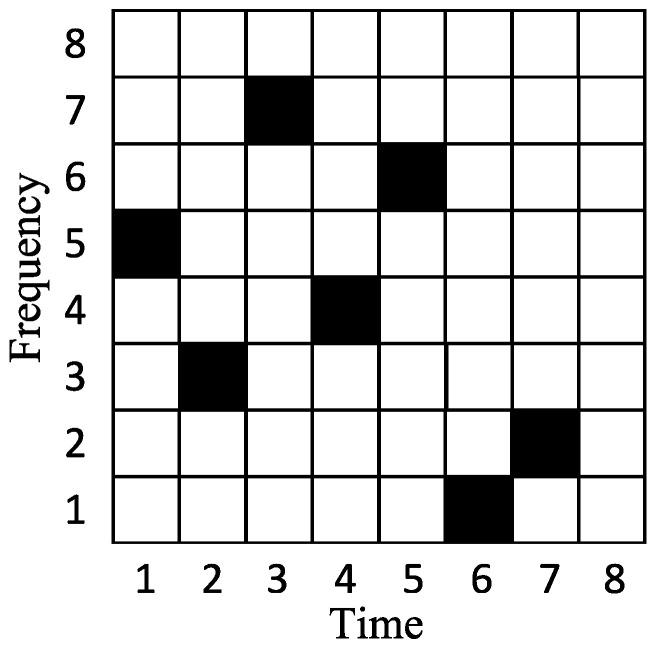
Golomb Costas array $C_{1}$.

Setting q=9, the Golomb Costas array is constructed as sequence {5, 3, 7, 4, 6, 1, 2}, based on Equation (3) and Table 1. Adding an empty column to the right and an empty row to the top of C forms $C_{1}$, which serves as the initial frequency hopping pattern for generating the family heads of the OFHPs, as shown in Figure 2.

Array $C_{1}$ shifts upward by 2, 4, and 6 rows, generating $C_{2}$, $C_{3}$, and $C_{4}$. These arrays act as family heads of frequency hopping patterns for each cell.

**Figure 3:**
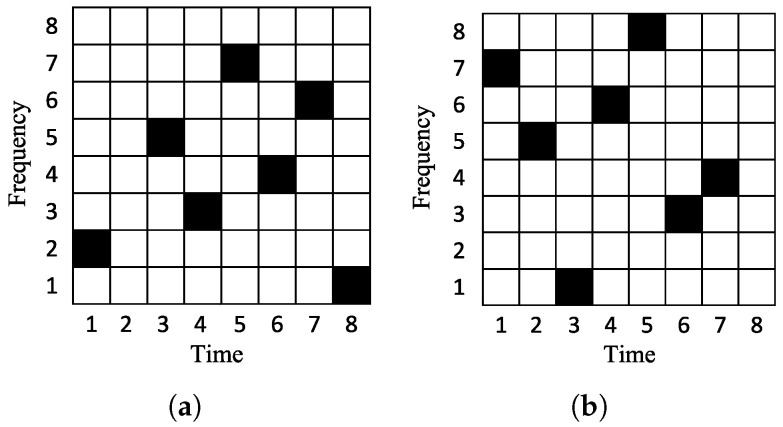
Optimal frequency hopping patterns. (**a**) Pattern 2 of family 1. (**b**) Pattern 1 of family 2.

Arrays $C_{1}$ and $C_{2}$ serve as the initial frequency hopping patterns for families 1 and 2. Shifting them left horizontally generates the OFHPs within each family. Figure 3a,b show representative patterns from families 1 and 2.

## 5. Simulation of Massive MIMO Systems Using Optimal Frequency Hopping Patterns

In a Massive MIMO system, assume $M_{1}=25$ users per cell, each with $M_{2}=4$ transmitting antennas. The cluster has $N_{1}=3$ cells, each base station with $N_{2}=20$ receiving antennas, assigning $N_{3}=1$ family of OFHPs per antenna. Based on Equation (9) and Equation (10), the system requires s=60 families, each containing $s_{1}=5$ frequency hopping patterns. Given the maximum normalized Doppler shift $d_{\max}=1$ and maximum multipath delay $\tau_{\max}=1$, the minimum Doppler distance is w=2, and the minimum time-delay distance is $w_{1}=2$. After constructing the Golomb Costas array, BPSK modulation is used to simulate the BER for a single cell. The received signal model is shown in Equation (11), and the BER calculation formula is given in Equation (12) [21].

(11) $$\begin{array}{cc} y_{u}\left(t\right) & =\sqrt{p}\cdot s_{u}\left(t\right)+\sum_{i\in I_{t}}\alpha_{i}\cdot s_{i}\left(t-\tau_{i}\right)\cdot \delta (f_{i}\left(t-\tau_{i}\right)\\ \; & \;+d_{i}-f_{u}\left(t\right))+n_{u}\left(t\right), \end{array}$$

where $y_{u}\left(t\right)$ denotes the received signal of user *u* at time *t*, $s_{u}\left(t\right)$ is the BPSK-modulated signal of user *u*, and $\alpha_{i}\in \left[0.25,0.45\right]$ represents the gain of the *i*-th multipath interference. The term $\tau_{i}$ denotes the delay of the *i*-th path, $d_{i}$ is the Doppler-induced frequency shift, and $f_{i}\left(t\right)$ is the hopping frequency of the interfering user (determined by FH(i,t)=1). The $f_{u}\left(t\right)$ is the hopping frequency of user *u*, and δ(·) is an indicator function that equals 1 when the frequencies match and 0 otherwise. $I_{t}$ is the set of all potential interferers at time *t*, and $n_{u}\left(t\right)∼\mathrm{CN}\left(0,\sigma^{2}\right)$ denotes the complex additive white Gaussian noise.

(12) $$\mathrm{BER}(\mathrm{SNR})=\frac{1}{u\cdot \left(n-\frac{n}{T}\right)}\sum_{j=1}^{u}\sum_{t\in A_{j}}1\left({\hat{b}}_{j}\left(t\right)\neq b_{j}\left(t\right)\right),$$

where *u* is the number of users, *n* is the total number of time slots per user, and *T* denotes the frequency hopping period. $A_{j}$ is the set of non-idle time slots for user *j*, $b_{j}\left(t\right)$ is the transmitted bit of user *j* at time *t*, and ${\hat{b}}_{j}\left(t\right)$ is the detected bit. The indicator function 1(·) equals 1 if an error occurs (i.e., ${\hat{b}}_{j}\left(t\right)\neq b_{j}\left(t\right)$), and 0 otherwise.

At the receiver, the signal $y_{u}\left(t\right)$ in Equation (11) is passed through a demodulator or detector to obtain the estimated bit ${\hat{b}}_{u}\left(t\right)$, which is then compared with the transmitted bit $b_{u}\left(t\right)$. Equation (12) computes the BER by counting the positions where ${\hat{b}}_{j}\left(t\right)\neq b_{j}\left(t\right)$ across all users and valid time indices, normalized by the total number of effective bits.

In the simulations, each user transmits $N=T\times 10^{5}$ BPSK-modulated bits, where *T* denotes the hopping period length, resulting in a total of U·N transmitted bits for a system with *U* users. The signal-to-noise ratio (SNR) is varied from 0 to 16 dB, with a step size of 1 dB. The channel noise is modeled as additive white Gaussian noise n∼CN(0,1), and the received signal is appropriately scaled according to the target SNR. A hard-decision detector is employed for BPSK demodulation, where transmitted bits are decided based on the sign of the real part of the received signal. The multipath delay is modeled as a discrete uniform random variable within $\left[1,\tau_{\max}\right]$, while the Doppler shift follows a discrete uniform distribution within $\left[-\nu_{\max},\nu_{\max}\right]$. The bit error rate (BER) is computed over all users and all transmitted information-bearing time slots (excluding idle slots defined by the hopping patterns). The results are averaged over all users and channel realizations under independent noise and fading conditions.

### 5.1. Simulation and Analysis of Multi-User Bit Error Rate

**Figure 4:**
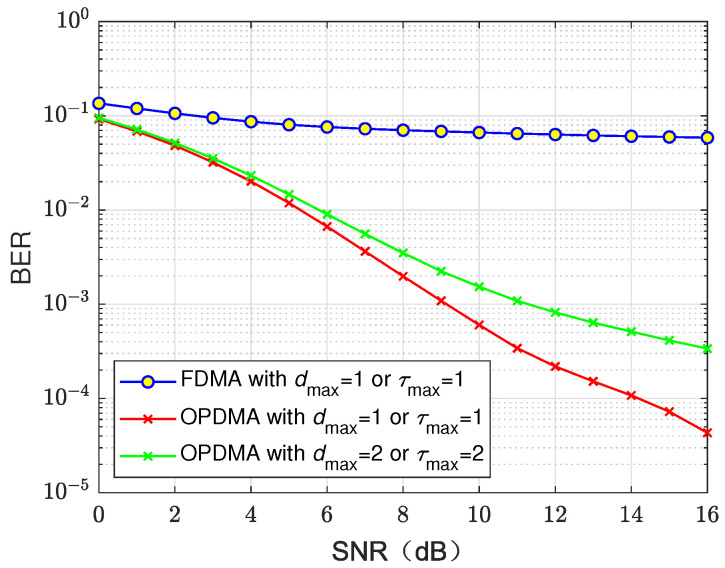
BER comparison for multiple users.

Figure 4 shows the BER performance in an AWGN multipath channel with 25 users per cell. Some of the results are presented in Table 2. In a multi-user environment, self-interference from multipath delays and Doppler shifts, along with inter-user interference, impacts communication quality. We simulated the BER for OPDMA and FDMA under $\tau_{\max}=1$ or $d_{\max}=1$ (i.e., $d_{\max}<w$ or $\tau_{\max}<w_{1}$), where OFHPs exhibit ideal autocorrelation and cross-correlation, and for OPDMA under $\tau_{\max}=2$ or $d_{\max}=2$ (i.e., $d_{\max}<w$ or $\tau_{\max}<w_{1}$), where cross-correlation increases. As shown in Figure 4, OPDMA achieves significantly lower BER than FDMA. However, when Doppler shift or delay is not smaller than the corresponding OFHPs distance, inter-user interference increases, raising BER.

### 5.2. Comparison, Simulation, and Analysis of Frequency Hopping Patterns Between OFHPs and the L-G Model

Since the optimal frequency hopping pattern arrays discussed above have a period of 127, the L-G model frequency hopping pattern arrays, based on the m-sequence, are set to a period of 15 (n=7). When the number of taps z=6, the number of frequency-hopping arrays meets the requirements, but the number of frequency points is $2^{6}=64$, which is relatively limited. In contrast, setting z=7 increases the frequency points to $2^{7}=128$, which is much closer to the 127 frequency points of the OFHPs. The next section compares the L-G model frequency hopping patterns for z=6 and z=7 with the OFHPs.

**Figure 5:**
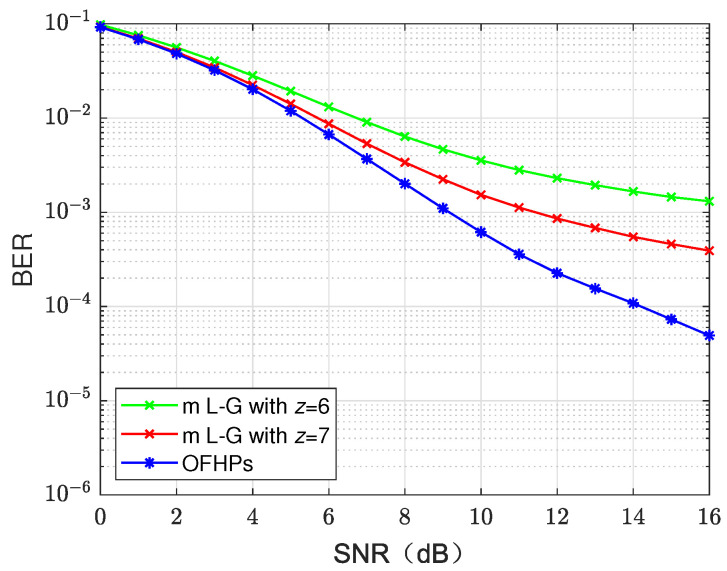
BER comparison of OFHPs and L-G model frequency hopping patterns.

Figure 5 presents the BER comparison for a 25-user cell, contrasting OFHPs with the L-G model frequency hopping patterns. Some of the results are presented in Table 3. When $d_{\max}<w$ and $\tau_{\max}<w_{1}$, OFHPs achieves a much lower BER than the L-G model patterns.

For OFHPs, these conditions ensure ideal cross-correlation between frequency hopping patterns. However, for L-G model patterns with z=6 and z=7, cross-correlation performance is weaker, resulting in a higher BER, as shown in Figure 5. The L-G model uses m-sequences for frequency hopping, which exhibit ideal one-dimensional autocorrelation. However, the frequency-hopping signals they control do not maintain ideal cross-correlation properties, limiting their effectiveness in mitigating interference. In contrast, the signals controlled by OFHPs exhibit ideal two-dimensional autocorrelation and cross-correlation properties, providing superior interference suppression and enhancing system performance. This demonstrates the stronger anti-interference ability of OFHPs, which, when designed to match the system’s maximum Doppler shift and multipath delay, surpasses the L-G model patterns, improving communication quality.

## 6. Conclusions and Future Work

In massive MIMO systems, multipath propagation, Doppler shifts, user-to-user interference, and pilot contamination can significantly degrade system performance. The proposed OPDMA technology demonstrates the capability to mitigate the effects of multipath propagation, Doppler shifts, and user interference through the use of two-dimensional orthogonal frequency hopping patterns.

Under the considered system conditions, OPDMA has the potential to reduce the reliance on equalization and channel estimation, which may simplify receiver design and reduce pilot resource usage, thereby helping alleviate pilot contamination. These characteristics suggest that OPDMA can serve as a promising low-complexity communication framework for supporting the practical deployment of massive MIMO systems while improving system robustness and spectral efficiency.

Future work will focus on evaluating the proposed OPDMA framework under more realistic communication environments, including standardized fading channel models, imperfect synchronization conditions, user mobility, channel estimation errors, and multi-cell pilot reuse scenarios, as well as hardware-oriented implementation and link-level/system-level validation of the proposed OPDMA framework.

## Figures and Tables

**Table 1:** Primitive elements and their powers in $GF(3^{2})$ ($f\left(x\right)=x^{2}+2x+2$).

α=x	β=x+2	ρ=2x	σ=2x+1
$\alpha^{2}=x+1$	$\beta^{2}=2x+2$	$\rho^{2}=x+1$	$\sigma^{2}=2x+2$
$\alpha^{3}=2x+1$	$\beta^{3}=2x$	$\rho^{3}=x+2$	$\sigma^{3}=x$
$\alpha^{4}=2$	$\beta^{4}=2$	$\rho^{4}=2$	$\sigma^{4}=2$
$\alpha^{5}=2x$	$\beta^{5}=2x+1$	$\rho^{5}=x$	$\sigma^{5}=x+2$
$\alpha^{6}=2x+2$	$\beta^{6}=x+1$	$\rho^{6}=2x+2$	$\sigma^{6}=x+1$
$\alpha^{7}=x+2$	$\beta^{7}=x$	$\rho^{7}=2x+1$	$\sigma^{7}=2x$
$\alpha^{8}=1$	$\beta^{8}=1$	$\rho^{8}=1$	$\sigma^{8}=1$

**Table 2:** BER comparison between FDMA and OPDMA under different $d_{\max}$ or $\tau_{\max}$.

SNR (dB)	FDMA ($d_{\max}=1$ or $\tau_{\max}=1$)	OPDMA ($d_{\max}=1$ or $\tau_{\max}=1$)	OPDMA ($d_{\max}=2$ or $\tau_{\max}=2$)
5	$8.8\times 10^{-2}$	$1.2\times 10^{-2}$	$1.5\times 10^{-2}$
10	$7.5\times 10^{-2}$	$6.0\times 10^{-4}$	$1.5\times 10^{-3}$
15	$6.5\times 10^{-2}$	$7.0\times 10^{-5}$	$4.0\times 10^{-4}$
16	$6.3\times 10^{-2}$	$5.0\times 10^{-5}$	$3.5\times 10^{-4}$

**Table 3:** BER comparison between OFHPs and m L-G patterns.

SNR (dB)	m L-G (z=6)	m L-G (z=7)	OFHPs
5	$2.0\times 10^{-2}$	$1.5\times 10^{-2}$	$1.2\times 10^{-2}$
10	$3.5\times 10^{-3}$	$1.5\times 10^{-3}$	$6.0\times 10^{-4}$
15	$1.5\times 10^{-3}$	$4.5\times 10^{-4}$	$7.0\times 10^{-5}$
16	$1.3\times 10^{-3}$	$3.8\times 10^{-4}$	$5.0\times 10^{-5}$

## Data Availability

Data is contained with the article. The original contributions presented in this study are included in the article. Further inquiries can be directed to the corresponding author(s).
